# The characteristics and influencing factors of fever in postoperative patients undergoing percutaneous nephrolithotomy: A retrospective analysis

**DOI:** 10.1097/MD.0000000000026485

**Published:** 2021-08-13

**Authors:** Ying Yu, Jieyu Pu, Tingting Wu, Li Hu

**Affiliations:** Department of Urology, Wuhan Central Hospital, Tongji Medical College, Huazhong University of Science and Technology, Wuhan, Hubei, China.

**Keywords:** care, fever, nursing, percutaneous nephrolithotomy, surgery, treatment, urine

## Abstract

Percutaneous nephrolithotomy (PCNL) is commonly used for the treatment of upper urinary calculi in clinical setting, and fever is a common complication after PCNL. It is necessary to evaluate the risk factors of fever in patients undergoing PCNL, to provide insights into the management of PCNL.

Patients who underwent PCNL in our hospital from January 2018 to August 2020 were included. The clinical data of postoperative fever and no fever patients were collected and assessed. Logistic regression analyses were conducted to analyze the risk factors for fever in patients undergoing PCNL.

A total of 276 patients undergoing PCNL were included, the incidence of postoperative fever for patients undergoing PCNL was 19.39%. No significant differences in the gender, body mass index, alcohol drinking, smoking, hypertension, hyperlipidemia, intraoperative blood infusion, length of hospital stay between fever patients, and no fever patients were found (all *P* > .05). There were significant differences in the age, diabetes, size of stones, duration of surgery between fever patients, and no fever patients (all *P* < .05). Age ≥60 years (odds ratio [OR] 2.143, 95% confidence interval [CI] 1.101∼3.264), diabetes (OR 2.218, 95% CI 1.176∼4.642), size of stone ≥2 cm (OR 1.428, 95%CI 1.104∼2.055), duration of surgery ≥100 minutes (OR 1.334, 95% CI 1.015∼1.923) were the risk factors for fever in patients with PCNL (all *P* < .05). *Escherichia coli* (48.44%), *Staphylococcus aureus* (18.75%), and *Candida albicans* (10.93%) were the top 3 pathogenic bacteria of urine culture.

Fever is one of the common complications after PCNL. Patients with high-risk factors should be given full attentions and take corresponding preventive measures targeted on risks.

## 1 Introduction

Urolithiasis is a common disease in urology, which produce great adverse influence on people's lives and health.^[1]^ As people's living environment, diet, exercise and other factors change, the incidence of urolithiasis in the world is also increasing year by year.^[2]^ Different countries have different prevalence of urinary stones with regards to different races, climates, socioeconomic conditions, education levels, eating habits, and other factors.^[3,4]^ According to reports,^[5,6]^ the prevalence of urinary stones in China is between 4.11% and 6.4%. The existence of stones will have many adverse effects on the human body. Previous studies^[7,8]^ have shown that stones are closely related to many common chronic diseases such as hypertension, diabetes, and chronic kidney disease, and so on. This will correspondingly increase the economic burden of medical and health care system. Therefore, the timely and effective treatment and management for urinary calculi is very important.

Percutaneous nephrolithotomy (PCNL) has gradually replaced open surgery as the first choice for the treatment of upper urinary calculi in clinical setting, but there are still complications such as bleeding, fever, sepsis, and so on.^[8,9]^ Among them, postoperative fever is the most common complication, and the incidence of postoperative fever can be as high as 21.0% to 39.8%.^[10]^ Previous studies^[11,12]^ have shown that postoperative fever is closely related to sepsis and septic shock. Therefore, for patients undergoing PCNL, it is very important to manage the patient's body temperature after surgery and take appropriate treatment in time. Therefore, we aimed to analyze the characteristics of patients undergoing PCNL, to investigate the potential risk factors of postoperative fever by logistic regression analyses, thereby providing evidence to the corresponding preventive strategies of postoperative fever in patients undergoing PCNL.

## 2 Methods

### 2.1 Ethical issues

Our study was a retrospective study design. In this study, all methods were performed in accordance with the relevant guidelines and regulations. This present study had been certified and approved by the Ethics Committee of Wuhan Central Hospital (0120916), and written informed consents were obtained from all included patients.

### 2.2 Patients

In this study, patients who underwent PCNL in our hospital from January 2018 to August 2020 were selected as the research population. All patients underwent preoperative urinary system color Doppler ultrasound, CT, intravenous pyelography (intravenous pyelography, IVP) imaging examinations to be diagnosed as kidney stones or upper ureteral stones.

### 2.3 Inclusion and exclusion criteria

The inclusion criteria of the patients in this study were: the age of patients was ≥18 years; the patient had no history of PCNL; patient did not have severe complications of heart, lung, and brain; patient did not have serious blood system disease; patients did not have urinary system tumors; patients were well informed and willing to participate in this study. Among them, all the patients received PCNL to establish a percutaneous renal channel for the first time. We excluded patients with tumors, hematological diseases, polycystic kidney disease, and renal failure.

### 2.4 Definitions

All the treatments were performed in accordance with the “Guidelines for the Diagnosis and Treatment of Urinary Surgery Diseases in China,”^[13,14]^ and the urine culture was selected from the clean mid-stage urine specimens of the patients who wake up in the morning. We quantitatively inoculated urine specimens on blood agar plates and MacConkey plates respectively, incubated in an incubator to do bacterial culture plus colony count. if there was no bacterial growth after prolonged incubation, the bacterial culture was considered negative. The specimens with positive results were taken from typical colonies for preliminary identification, and bacterial identification was performed on the strains. Considering that the postoperative body temperature is <38°C, most of the mild fever was associated with reaction caused by the operation itself did not require special treatment. In this study, postoperative body temperature >38°C was defined as postoperative fever.

### 2.5 PCNL

For diabetic patients, we would control the patient's blood glucose level within the normal range before surgery. The patient received general anesthesia, and the stone cut position was taken. The F5 ureteral catheter was inserted into the renal pelvis via a cystoscope. A 0.9% sodium chloride solution was continuously injected through the ureteral catheter. Then the patient changed to the prone position, we selected 10–12 cm next to the spine and 12 behind the back of the axillary line as the puncture point, and punctured into the target renal calyx under ultrasound guidance. Then we used the EMS ultrasound system for lithotripsy. After success, the double J tube was routinely indwelled to fix the nephrostomy tube to the skin.

### 2.6 Statistical analysis

SPSS 22.0 statistical software was applied to process collected data. The mean of each parameter was expressed as mean ± standard deviation. Comparisons between the means were compared using *t* tests, *χ*^2^ test were conducted for percentage data. Logistic regression analyses were conducted for analyzing the risk factors for fever in patients undergoing PCNL. In this study, *P* < .05 was considered as statistically significant.

## 3 Results

### 3.1 Patients’ characteristics

A total of 276 patients undergoing PCNL were included, of whom 46 patients had postoperative fever, the incidence of postoperative fever for patients undergoing PCNL was 19.39%. As showed in Table 1, no significant differences in the gender, body mass index, alcohol drinking, smoking, hypertension, hyperlipidemia, intraoperative blood infusion, length of hospital stay between fever patients, and no fever patients were found (all *P* > .05), and there were significant differences in the age, diabetes, size of stones, duration of surgery between fever patients and no fever patients (all *P* < .05).

**Table 1:** The characteristics of included patients.

Variables	Fever group (n = 48)	No fever group (n = 228)	*t*/*χ*^2^	*P*
Male/female	35/13	171/57	1.311	.087
Age, y	62.17 ± 6.24	57. 13 ± 9.18	1.419	.015
BMI, kg/m^2^	24.43 ± 1.89	24.15 ± 3.04	1.274	.072
Alcohol drinking	29 (60.42%)	130 (57.02%)	1.104	.089
Smoking	21 (43.75%)	92 (40.35%)	1.132	.107
Hypertension	27 (56.25%)	121 (53.07%)	1.122	.091
Diabetes	24 (50%)	74 (32.46%)	1.014	.003
Hyperlipidemia	8 (16.67%)	31 (13.60%)	1.316	.095
Size of stone, cm	1.94 ± 0.87	1.28 ± 0.51	1.039	.042
Duration of surgery, min	121.07 ± 28.55	98.42 ± 30.29	1.162	.034
Intraoperative blood infusion	13 (27.08%)	55 (24.12%)	1.288	.071
Length of hospital stay, days	4.57 ± 1.21	4.12 ± 1.08	1.107	.105

### 3.2 The risk factors for fever in patients with PCNL

The variable assignments of multivariate logistic regressions were presented in Table 2. As indicated in Table 3, age ≥ 60 years (odds ratio [OR] 2.143, 95% confidence interval [CI] 1.101∼3.264), diabetes (OR 2.218, 95% CI 1.176∼4.642), size of stone ≥2 cm (OR 1.428, 95% CI 1.104∼2.055), duration of surgery ≥100 minutes (OR 1.334, 95% CI 1.015∼1.923) were the risk factors for fever in patients with PCNL (all *P* < .05).

**Table 2:** The variable assignment of multivariate logistic regression.

Factors	Variables	Assignment
Fever	Y	yes = 1, no = 2
Age, y	X_1_	≥60 = 1, <60 = 2
Diabetes	X_2_	yes = 1, no = 2
Size of stone, cm	X_3_	≥2 = 1, <2 = 2
Duration of surgery, min	X_4_	≥100 = 1, <100 = 2

**Table 3:** Logistic regression analysis on the risk factors for fever in patients with PCNL.

Variables	β	SE	OR	95% CI	*P*
Age ≥60 y	0.118	0.219	2.143	1.101∼3.264	.028
Diabetes	0.102	0.283	2.218	1.176∼4.642	.032
Size of stone ≥2 cm	0.114	0.113	1.428	1.104∼2.055	.017
Duration of surgery ≥100 min	0.173	0.306	1.334	1.015∼1.923	.043

### 3.3 Urine culture

Of the 48 patients with fever in this present study, a total of 64 cases of bacteria were detected. As presented Table 4, *Escherichia Coli* (48.44%), *Staphylococcus aureus* (18.75%), and *Candida albicans* (10.93%) were the top 3 pathogenic bacteria in urine culture.

**Table 4:** The pathogenic bacteria in urine culture.

Bacteria	Cases (n = 64)	Percent
*Escherichia Coli*	31	48.44%
*Staphylococcus aureus*	12	18.75%
*Candida albicans*	7	10.93%
*Klebsiella pneumoniae*	6	9.38%
*Pseudomonas aeruginosa*	4	6.25%
*Enterococcus faecium*	3	4.69%
*Comamonas testosterone*	1	1.56%

## 4 Discussions

Clinically, postoperative fever is common in patients undergoing PCNL, and the probability of postoperative fever can be as high as 39.8%.^[15]^ Postoperative fever is clinically closely related to sepsis and septic shock.^[16]^ If serious complications occur, it may endanger the life and health of patients.^[17]^ Thus we should pay attention to the fluctuation of postoperative body temperature in patients undergoing PCNL surgery, and adopt corresponding treatment plans in time. Urinary tract infections are commonly seen in patients after PCNL. Once infections occur, sensitive antibiotics should be used for anti-infection treatment based on drug sensitivity tests. However, studies^[18,19]^ have shown that infections most often occur within 6 hours after surgery, and blood bacteria culture results are generally available about 4 days, and urine bacteria culture results are generally available in about 2 days. The results of this present study have found that the incidence of postoperative fever for patients with PCNL is 19.39%, and age ≥60 years, diabetes, size of stone ≥2 cm, duration of surgery ≥100 minutes is the risk factors for fever in patients with PCNL, it is clinically necessary to adopt early prevention and response strategies for these risk factors.

The global population is now tending to be aging. Demographic statistics reports have indicated that the world's elderly population (age > 60 years’ old) will increase from 841 million in 2013 to >2 billion in 2050.^[20]^ Among all stone patients, the elderly has accounted for a total of 10% to 12%, and due to changes in the metabolic characteristics of the elderly,^[21]^ the composition of their stones is different from that of the younger population. The proportion of uric acid stones in elderly stone patients is much higher, so it is necessary to analyze the postoperative complications of PCNL in elderly patients. In this study, elderly patients are more likely to have fever after surgery. This is related to the fact that the elderly have more comorbidities and poor physical function.^[22]^ The stones in the elderly patients were mostly cast stones, which may increase the difficulty of the operation and the duration of the operation, resulting in greater damage to the renal pelvic mucosa during lithotripsy, and it also increases the absorption of the perfusion fluid.^[23]^ In addition, elderly patients have weaker resistance to Staphylococcal toxins, so they are more likely to have fever after surgery.^[24]^

The patients with size of stone ≥2 cm and duration of surgery ≥100 minutes have a higher incidence of postoperative fever in this study. It has been reported that large stones such as staghorn stones, among others, usually cause upper urinary tract obstruction. Stones are usually formed by the long-term deposition of urine sediment.^[25]^ When the urinary tract is obstructed, the bacteria on the surface of the stones are difficult to excrete outside the body and grow in large numbers in the renal pelvis.^[26]^ When the stones are crushed, the urinary tract infection-causing bacteria and toxins contained in the stone will be released in large quantities, and enter the blood circulation through the damaged renal pelvic mucosa, causing infection and fever.^[27]^ In severe cases, it will cause sepsis and septic shock, which will threaten the life of the patient.^[28]^ In addition, larger stones often complicate the operation.^[29]^ Due to the need for more operations, the contact between the nephroscope and the renal pelvic mucosa increases during the lithotripsy process, and the probability of damage to the renal pelvic mucosa will be greater.^[30]^ And the chance of pulmonary infection caused by toxin backflow into the blood is greatly increased.^[31]^ Excessive operation time will increase the absorption of intraoperative perfusion fluid, which may increase the chance of bacteria such as *Pseudomonas aeruginosa* and *Clostridium difficile* toxins entering the human blood, which greatly increases the chance of infection after surgery.^[32,33]^ It has been reported that that patients with larger stones and preoperative urinary tract infections had a higher risk of systemic inflammatory response syndrome and fever after surgery,^[34]^ which is consistent with our findings. Furthermore, obstructive nephropathy is an evolving disease in which the renal damage continues even after relief of the obstruction,^[35]^ which may be closely associated with the postoperative urinary infection.

In this study, diabetes is an independent risk factor for fever after PCNL. It has been reported that the risk of kidney stones in diabetic patients is higher than that of other populations. There have been many studies^[23,36]^ on the relationship between kidney stones and diabetes, and it is generally believed that the formation of various types of stones is strongly influenced by urinary pH. An alkaline pH favors the crystallization of calcium- and phosphate-containing stones, whereas an acidic urine pH promotes uric acid or cystine stones.^[37]^ In diabetic patients, uric acid stones are more common, and the proportion is about 30.40%, whereas uric acid stones in other populations are about 5.10%.^[38,39]^ Insulin resistance will change the electrolytes in the urine, leading to acidification of the urine.^[40]^ It can directly act on the renal tubules to reduce ammonia production and increase sodium reabsorption, which are important risk factors for the formation of stones.^[41]^ In this study, patients with diabetes have a higher incidence of fever after PCNL. There are reports^[42,43]^ indicating that people with diabetes also have higher chances of kidney stone formation, this may be related to the reduced immunity of diabetes patients. Diabetic patients with poor blood sugar control often have both phagocytosis, intracellular sterilization, and cellular deficiencies in various defense functions such as reduced immunity make diabetic patients prone to urinary tract infections.^[44]^

Several limitations in this present study must be concerned. First, the sample size was small is this study, it might be underpowered to detect the group differences, more studies with larger sample size on this issue are needed in the future. Secondly, our study was a retrospective design, many factors that might potentially influence the fever for patients with PCNL such as antibiotics use, stress level, among others, could be included for data analysis. And the preoperative status of urinary tract infection in the patient population which might be an important risk factor as it might lead to sepsis and fever post-operative; we could not analyze this issue limited by insufficient data. Besides, details on the severity of diabetes needs to be further studied in the fever versus non-fever patients. Therefore, future studies with prospective rigorously design in different areas are needed to further elucidate the influencing factors of fever after PCNL.

## 5 Conclusions

In summary, the postoperative fever for patients with PCNL very common, and age ≥60 years, diabetes, size of stone ≥2 cm, duration of surgery ≥100 minutes are the risk factors for fever in patients with PCNL. Therefore, for patients undergoing PCNL, targeted measures should be taken for their risk factors, such as preventive use of antibiotics before surgery based on the results of *Escherichia coli*, *Staphylococcus aureus*, and *Candida albicans* were the most commonly seen pathogenic bacteria in urine culture. Besides, it is necessary to strictly control the duration of surgery. The second-stage surgical treatment may be appropriate for those who cannot complete the lithotripsy in the first stage. Furthermore, the diabetic patients need to regulate their blood sugar before surge ry to reduce the chance of postoperative infection. Some clinicians tend to ignore those risk factors during diagnosis and treatment, which leads to higher fever incidence after PCNL. Therefore, we should pay attention to those factors and take counteractive measures in order to make PCNL safer and more effective.

## Author contributions

Y Y, L H designed research; Y Y, J P, T W, L H conducted research; Y Y, J P analyzed data; L H wrote the first draft of manuscript; L H had primary responsibility for final content. All authors read and approved the final manuscript.

**Data curation:** Ying Yu.

**Formal analysis:** Ying Yu.

**Funding acquisition:** Li Hu.

**Investigation:** Ying Yu, Jieyu Pu, Tingting Wu, Li Hu.

**Methodology:** Tingting Wu, Li Hu.

**Project administration:** Ying Yu.

**Resources:** Jieyu Pu.

**Software:** Ying Yu, Tingting Wu, Li Hu.

**Supervision:** Jieyu Pu.

**Visualization:** Li Hu.

**Writing – original draft:** Li Hu.
